# Factors affecting clinical outcomes after IVF-ET for infertile young patients with ovarian endometrioma: A 5-year retrospective cohort study

**DOI:** 10.1097/MD.0000000000029793

**Published:** 2022-06-30

**Authors:** Feng Zhou, Fanxuan Zhao, Xiaoying Jin, Chao Li, Songying Zhang

**Affiliations:** a Assisted Reproduction Unit, Department of Obstetrics and Gynecology, Sir Run Shaw Hospital, Zhejiang University School of Medicine; Key Laboratory of Reproductive Dysfunction Management of Zhejiang Province, Hangzhou 310016, China.

**Keywords:** endometriosis, infertility, IVF-ET, ovarian endometrioma, ovarian reserve function

## Abstract

This study aimed to compare ovarian reserve function and outcomes after in vitro fertilization and embryo transfer (IVF-ET) for young women with pelvic endometriosis with or without ovarian endometrioma. We explored the main factors influencing pregnancy outcomes in young patients with endometrioma.

A total of 619 patients ≤38 years of age who underwent IVF-ET in our reproductive center between January 2011 and December 2015 were recruited. Among these patients, 398 had pelvic endometriosis with ovarian endometrioma and 221 had pelvic endometriosis without ovarian endometrioma. Patients underwent ovulation induction during IVF-ET. The general conditions and clinical outcomes of IVF-ET treatment were compared. Key factors affecting the success of IVF-ET treatment for endometriomas were analyzed.

During IVF-ET treatment, the numbers of retrieved oocytes and 2-pronuclei (2PN) embryos in all age groups (*P* < .01), and the number of 2PN high-quality embryos in patients under 30 years of age was lower in the pelvic endometriosis with ovarian endometrioma group than in the pelvic endometriosis alone group (*P* < .05). Logistic regression analysis showed the number of antral follicles, basal follicle-stimulating hormone (bFSH) levels, number of oocytes, number of 2PN embryos, and number of 2PN high-quality embryos were significantly related to the successful outcome of IVF-ET. Among these, the number of 2PN high-quality embryos was the only independent predictive factor.

Ovarian endometrioma significantly impairs ovarian reserve function and ultimately affects the therapeutic efficacy of IVF-ET. Obtaining more 2PN high-quality embryos was important for IVF-ET treatment of young patients with ovarian endometriomas.

## 1. Introduction

Endometriosis (EMs) is closely related to infertility. Approximately 30% to 50% of patients with EMs exhibit infertility,^[1]^ and women with infertility are 6 to 8 times more likely to have EMs than fertile women.^[2]^ Ovarian endometrioma is the most common manifestation of EMs^[3]^; 30% to 40% of patients with EMs have ovarian endometrioma.^[4]^ Studies have reported that 55% of patients with ovarian endometrioma have fibrosis in the ovarian cortex, leading to a reduction in ovarian function and follicles.^[5]^ In vitro fertilization and embryo transfer (IVF-ET) can effectively improve the pregnancy rate of infertile patients with EMs.^[6]^ However, pregnancy outcomes after IVF-ET were significantly lower for patients with EMs than those of other patients.^[7]^

This study retrospectively analyzed the pregnancy outcomes of young patients (≤38 years) with EMs who were treated with IVF-ET at our reproduction center between January 2011 and December 2015. The general conditions and IVF outcomes of patients with EMs and ovarian endometrioma were compared with those of patients with pelvic EMs without ovarian endometrioma. The main factors affecting pregnancy outcomes in patients with ovarian endometrioma were studied to provide a reference for clinical therapy.

## 2. Methods

### 2.1. Ethics statement

This study was approved by the Institutional Ethical Review Board of Sir Run Run Shaw Hospital, Affiliated Hospital of the College of Medicine, Zhejiang University.

### 2.2. Study design

A total of 619 patients with EMs were included in our reproductive center from January 2011 to December 2015, including 398 patients with pelvic EMs with ovarian endometrioma and 221 with pelvic EMs without ovarian endometrioma. The inclusion criteria were as follows: age ≤38 years, ovarian endometrioma was diagnosed from surgical samples and pathology results indicated an EM cyst, without malignant or borderline lesions. If the patient had no history of other surgery, ovarian endometriomas was diagnosed by an experienced B-ultrasound doctor. The diagnosis of pelvic EMs requires the detection of typical endoscopic lesions during laparoscopy. All ET cycles were performed using the ovulation stimulation protocol, and pregnancy outcomes were calculated after the first ET of the cycles. The exclusion criteria were chromosomal abnormalities, uterine leiomyoma ≥3 cm or compression of the endometrium, uterine adenomyosis, intrauterine adhesion, other congenital genital dysplasia and deformities, other benign and malignant ovarian tumors, history of abdominal surgery except EMs, and serious organ diseases, such as heart, liver, brain, and lung disease. All cycles were categorized into 2 groups based on patient age: ≤30 years (group A), 31 to 38 years (Group B). Every patient, and nearly all couples, acknowledged that they fully understood the protocol and then signed the informed consent form.

### 2.3. Assessment of embryo quality

The embryo culture system was a G-1.3 series sequential culture system. Embryo transfer and freezing were performed on the third day after oocyte retrieval. Embryo quality assessment was based on the morphology and rate of development in culture. Embryos with a normal cleavage rate (3–4 cells on the second day and 6–8 cells on the third day) and <20% fragmentation were defined as “high quality.”^[8]^

### 2.4. Pregnancy assessment

Successful pregnancy was confirmed by the detection of an increased serum β-human chorionic gonadotropin concentration 12 to 14 days after ET. Clinical pregnancy was defined as the observation of a gestational sac with or without a fetal heartbeat on ultrasound evaluation on the 35th day after ET. The number of sacs was defined as the number of implantations.

### 2.5. Statistical analysis

All statistical calculations were performed using SPSS software (version 20.0; SPSS Inc., Chicago, IL). Quantitative variables are expressed as mean ± standard deviation (X ± SD), and Student t-test was used. Qualitative data were statistically analyzed using the Chi-squared test. Fisher exact probability method was used when the theoretical frequency was <1. Univariate logistic analysis and nonconditional multivariate logistic regression analysis were used for screening out parameters that may be meaningful for the IVF-ET treatment outcome in patients with ovarian endometrioma. Statistical significance was set at *P* <.05.

## 3. Results

### 3.1. Comparison of general conditions of ovarian endometrioma and pelvic EMs

A total of 619 patients with EMs and receiving IVF-ET between January 2011 and December 2015 were included in this study. A total of 398 patients with pelvic EMs with ovarian endometrioma were included (≤30 years: 179 cases, 31–38 years: 219 cases). There were 221 cases of pelvic EMs without ovarian endometrioma (≤30 years: 90 cases; 31–38 years: 131 cases). The mean age of the 2 groups was not significantly different. The number of antral follicles in patients with ovarian endometrioma was lower than that in patients with pelvic EMs alone (*P* < .01). It was found that bFSH levels were similar in all patients under 30 years of age, while in patients between 30 and 38 years of age, the levels of bFSH were higher in patients with ovarian endometrioma than those in patients with EMs but without ovarian endometrioma (*P* < .05) (Table 1).

**Table 1:** Comparison of general conditions in patients pelvic endometriosis with or without ovarian endometrioma.

	Ovarian endometrioma			Pelvic endometriosis		
	≤30 yrs	31–38yrs	Total	≤30 yrs	31–38yrs	Total
Cases	179	219	398	90	131	221
Age	28.1 ± 1.6	33.2 ± 2.0	30.9 ± 3.2	28.0 ± 2.1	33.2 ± 2.0	31.1 ± 3.2
Duration of infertility (yrs)	2.5 ± 1.5	3.9 ± 2.5	3.3 ± 2.2	3.2 ± 1.7*	4.8 ± 2.5*	4.1 ± 2.3*
bFSH	7.8 ± 3.1	8.3 ± 3.8	8.1 ± 3.5	7.4 ± 2.5	7.6 ± 2.4†	7.5 ± 2.4†
Number of antral follicle	7.0 ± 3.3	6.7 ± 3.2	6.8 ± 3.2	9.9 ± 3.9*	8.3 ± 3.5*	9.0 ± 3.7*
Total Gn (IU)	2155.4 ± 876.1	2308.3 ± 1781.5	2239.5 ± 1446.6	2001.8 ± 578.9	2072.9 ± 725.3	2044.0 ± 669.1†
Days of Gn duration	9.2 ± 2.6	9.2 ± 2.4	9.2 ± 2.5	9.2 ± 1.7	9.0 ± 2.1	9.1 ± 2.0

Comparison between pelvic endometriosis with or without ovarian endometrioma in the same age group. Parameters were all normally distributed, and a t-test was used.

bFSH = basal follicle-stimulating hormone, Gn = gonadotropins.

* *P* < .01.

† *P* < .05.

**Table 2:** Comparison of in vitro fertilization embryo transfer treatment outcomes in patients with pelvic endometriosis with or without ovarian endometrioma.

	Ovarian endometrioma			Pelvic endometriosis		
	≤30 yrs (179 cases)	31–38 yrs (219 cases)	Total (398 cases)	≤30 yrs (90 cases)	31–38 yrs (131 cases)	Total (221 cases)
Number of retrieved oocytes	8.2 ± 6.3	6.8 ± 5.4	7.4 ± 5.9	12.1 ± 6.9*	8.8 ± 6.0*	10.1 ± 6.5*
Fertilization rate	5.4 ± 4.9	4.6 ± 4.0	4.9 ± 4.4	8.1 ± 5.3*	5.8 ± 4.2*	6.7 ± 4.8*
2PN fertilization rate	66.2%	66.6%	66.4%	66.7%	66.5%	66.6%
	(968/1462)	(997/1497)	(1965/2959)	(728/1092)	(762/1146)	(1490/2238)
Cleavage rate	97.4%	98.4%	97.9%	97.8%	98.7%	98.3%
	(943/968)	(981/997)	(1924/1965)	(712/728)	(752/762)	(1464/1490)
Number of 2PN embryos	5.3 ± 4.8	4.5 ± 3.9	4.8 ± 4.4	7.9 ± 5.3*	5.7 ± 4.2*	6.6 ± 4.8*
Number of 2PN high-quality embryos	2.5 ± 3.0	2.2 ± 2.4	2.3 ± 2.7	3.6 ± 3.6†	2.5 ± 2.5	3.0 ± 3.0*
2PN high-quality embryos rate	47.6%	47.9%	47.8%	45.5%	43.9%	44.7%
	(449/943)	(470/981)	(919/1924)	(324/712)	(330/752)	(654/1464)
No embryo transfer ratio	14.0%	11.9%	12.8%	2.2%*	7.6%	5.4%*
	(25/179)	(26/219)	(51/398)	(2/90)	(10/131)	(12/221)
Number of embryo transfer	1.9 ± 0.4	1.7 ± 0.4	1.8 ± 0.4	1.8 ± 0.4	1.8 ± 0.4	1.8 ± 0.4
High-quality embryos transfer rate	72.3% (206/285)	68.8% (232/337)	70.4% (438/622)	71.7% (114/159)	66.5% (147/221)	68.7% (261/380)
Embryo implantation rate	42.5% (121/285)	40.1% (135/337)	41.2% (256/622)	37.1% (59/159)	37.1% (82/221)	37.1% (141/380)
Clinical pregnancy rate	57.8% (89/154)	57.5% (111/193)	57.6% (200/347)	56.8% (50/88)	57.0% (69/121)	56.9% (119/209)
Abortion rate	4.5% (4/89)	12.6% (14/111)	9.0% (18/200)	10% (5/50)	11.6% (8/69)	10.9% (13/119)

Comparison between patients with pelvic endometriosis/ovarian endometrioma and pelvic endometriosis alone in the same age group. The Chi-square test was used, and the Fisher exact probability method was used when the theoretical frequency was <1. Fertilization rate is the number of fertilized oocytes/number of retrieved oocytes; Cleavage rate is the number of embryos/number of fertilized oocytes; 2-pronuclei (2PN) high-quality embryo rate is the number of 2PN high-quality embryos/number of total embryos; Embryo implantation rate is the number of gestational sacs/number of transferred embryos; and Clinical pregnancy rate is the number of pregnancy cycles/number of transfer cycles.

PN = pronucleus.

* *P* < .01.

† *P* < .05.

### 3.2. Comparison of outcomes of IVF-ET treatment between patients with EMs with or without ovarian endometrioma

During IVF-ET treatment, the number of retrieved oocytes and 2-pronuclei stage (2PN) embryos in all age groups (*P* < .01), and the number of 2PN high-quality embryos in the ≤30 years group (*P* < .05) was lower in patients with ovarian endometrioma than those in patients with pelvic EMs alone. The rates of normal fertility, cleavage, 2PN high-quality embryos, embryo implantation, clinical pregnancy, and abortion between the 2 groups were considered similar (Table 2).

**Table 3:** Analysis of factors affecting clinical outcomes in patients with ovarian endometrioma.

	Success group	Failure group	*P* value
Cases	200	198	
Age	30.8 ± 3.2	31.0 ± 3.1	.468
Duration of infertility (yrs)	3.1 ± 2.2	3.5 ± 2.2	.130
Size of cysts	4.0 ± 2.1	4.0 ± 2.0	.959
Endometrioma surgery rate before IVF	96.5% (193/200)	94.4% (187/198)	.324
Endometrioma exist rate during IVF	24.5% (49/200)	22.7% (45/198)	.677
Number of antral follicle	7.4 ± 3.2	6.2 ± 3.2	<.001
bFSH	7.7 ± 2.5	8.4 ± 4.2	.039
Total Gn (IU)	2328.9 ± 1841.3	2149.3 ± 881.1	.216
Days of Gn duration	9.3 ± 2.3	9.1 ± 2.7	.282
Number of oocytes	8.6 ± 6.2	6.3 ± 5.3	<.001
Number of 2PN embryos	5.9 ± 4.7	3.9 ± 3.9	<.001
Number of 2PN high-quality embryos	3.2 ± 3.1	1.4 ± 1.8	<.001

Parameters were all normally distributed, and a t-test was used. Endometrioma surgery rate: the number of patients undergoing ovarian endometrioma surgery/ number of total patients; Endometrioma exist rate: the number of patients undergoing IVF treatment in the presence of ovarian endometrioma/number of total patients.

bFSH = basal follicle-stimulating hormone, Gn = gonadotropins, IVF = in vitro fertilization, PN = pronucleus.

**Table 4:** Results of univariate logistic analysis of influential factors of in vitro fertilization embryo transfer treatment outcomes.

Factors	*P* value	OR value
Age, yrs	.467	0.977
Duration of infertility (yrs)	.131	0.933
Number of antral follicle	<.001	1.137
bFSH	.044	0.937
Total Gn (IU)	.238	1.0
Days of Gn duration	.282	1.045
Number of oocytes	<.001	1.075
Number of 2PN embryos	<.001	1.135
Number of 2PN high-quality embryos	<.001	1.456

bFSH = basal follicle-stimulating hormone, Gn = gonadotropins, IVF = in vitro fertilization, PN = pronucleus.

**Table 5:** Results of influential factors of in vitro fertilization embryo transfer treatment outcomes of nonconditional multivariate logistic regression analysis.

Independent variable	Regression coefficients	Standard error	Wald value	*P* value	OR value	95% CI	
						Lower	Upper
Number of antral follicle	0.045	0.045	1.127	.289	1.049	0.869	1.053
bFSH	−0.034	0.035	0.94	.332	0.967	0.961	1.145
Number of oocytes	−0.048	0.049	0.966	.326	0.953	0.903	1.035
Number of 2PN embryos	−0.009	0.071	0.016	.9	0.991	0.866	1.049
Number of 2PN high-quality embryos	0.434	0.089	23.689	<.001	1.544	1.296	1.839

bFSH = basal follicle-stimulating hormone, PN = pronucleus.

### 3.3. Analysis of factors affecting clinical outcomes in patients with ovarian endometrioma

After undergoing IVF-ET, patients with ovarian endometrioma who became pregnant after the first ET had more antral follicles, retrieved oocytes, and 2PN embryos (*P* < .01), and had lower bFSH (*P* < .05) compared with patients with failure outcomes (including patients who were not pregnant after the first ET or who did not have embryos for transfer). There were no differences in age, duration of infertility, rate of ovarian endometrioma surgery (endometrioma surgery rate) before IVF, and rate of IVF treatment in the presence of ovarian endometrioma (endometrioma exist rate) (Table 3). The correlation between parameters and treatment success was determined by univariate logistic analysis. The results showed that years of infertility, bFSH, the number of retrieved oocytes, 2PN embryos, and 2PN high-quality embryos were related to successful outcomes (*P* < .05) (Table 4). Nonconditional multivariate logistic regression analysis was used with the above 5 significant factors. The number of 2PN high-quality embryos was the only independent predictive factor for clinical outcomes of IVF-ET, and the odds ratio value was 1.544 (Table 5).

## 4. Discussion

### 4.1. The mechanisms of EMs-induced infertility

In recent years, the incidence of EMs, which is closely associated with female infertility, has increased.^[9]^ Pathologically, patients with EMs commonly exhibit severe pelvic adhesions and a disordered pelvic structure. Under pathological conditions, elevated levels of prostaglandins are generated from intra-abdominal ectopic lesions and released into the peritoneal fluid, affecting the normal peristalsis of fallopian tubes and obstructing the collection of oocytes.^[10]^ In addition, the follicular structure of ovarian tissues can be damaged around ovarian endometrioma, triggering follicular maldevelopment, decreased oocyte and embryo quality,^[11]^ and ovarian dysfunction.^[12]^ Research has increasingly shown that endometrial receptivity in patients with EMs is significantly decreased and accompanied by implantation failure and infertility.^[13]^

Our previous work concluded that the rates of embryo implantation and clinical pregnancy were significantly lower in patients with EMs who received IVF/intracytoplasmic sperm injection treatment than in other patients.^[7]^ In this study, we mainly recruited patients under 38 years of age to eliminate age-related factors, based on the fact that older patients maintain a low pregnancy rate even after IVF/intracytoplasmic sperm injection treatment. The present study found that promising clinical outcomes for patients with EMs after the first transfer included a clinical pregnancy rate of 51.5% (319/619) and an abortion rate of 9.7% (31/319).

### 4.2. Comparisons between ovarian endometrioma with pelvic EMs and pelvic EMs alone after IVF treatment

Our study indicated that compared with pelvic EMs alone, young patients also exhibiting ovarian endometrioma were characterized by having fewer antral follicles and higher bFSH levels, suggesting that the ovarian function was significantly lower for cases with ovarian endometrioma than those with pelvic EMs alone. In addition, the number of retrieved oocytes and 2PN embryos from patients with ovarian endometrioma were markedly lower than those in patients with pelvic EMs alone, whereas the fertilization and high-quality embryo rates were similar between the 2 groups, suggesting that the effects of EMs on oocyte and embryo quality were independent of ovarian endometrioma. The clinical outcomes implied that patients with ovarian endometrioma were characterized by a higher rate of nontransferrable embryos, especially for patients aged ≤30 years, whereas similar rates of clinical pregnancy, embryo implantation, and abortion were found between the 2 groups for patients with transferrable embryos. These findings indicate that ovarian endometriomas may greatly influence ovarian function and restrict oocyte retrieval. Notably, the effects of ovarian endometriomas on endometrial receptivity and the quality of oocytes and embryos were similar to those of pelvic EMs.

Currently, whether surgical treatment or the ovarian endometrioma cyst itself is more harmful for ovarian function remains under discussion. An earlier study suggested that compared with other benign ovarian cysts, such as teratoma and benign cystadenoma, ovarian endometrioma affects the number of follicles in the ovarian cortex.^[14]^ Somigliana et al^[15]^ found that ovarian endometrioma could impede the distribution of ovarian blood vessels, thus reducing ovarian function. Schubert et al^[16]^ found that fibrosis occurred during ovarian endometrioma, but not during ovarian serous cyst or cortical cysts. Coccia et al^[17]^ also found that patients with bilateral ovarian endometrioma experienced early menopause postoperatively. Also, there appears to be a pseudocapsule surrounding ovarian endometriomas, which, when surgically excised, may remove part of the normal ovarian tissues. Local inflammation caused by surgery and vascular remodeling following electrocoagulation partially influences ovarian function. The rate of premature ovarian failure after cystectomy was reported as 2.4%.^[18]^ These results showed that both ovarian endometrioma and surgery influence ovarian function.

When the diameter of the ovarian endometrioma is >4 cm, it easily results in cyst rupture, infection, and follicular fluid contamination during the operation of oocyte collection. Olivennes^[19]^ proposed that surgery treatment before IVF could improve its success rate, especially in women under the age of 35, but repeated ovarian endometrioma surgery would affect ovarian function. Moreover, many gynecologists have suggested that ovarian endometriomas with a diameter ≥3 cm should be surgically removed because they would affect the measurement of follicles and oocyte retrieval.^[20–22]^ The European Society for Human Reproduction and Embryology also recommended laparoscopic ovarian endometrioma surgery when the diameter of the cyst was >4 cm.^[23]^ Therefore, it is important for patients with ovarian endometrioma to choose the best therapy after careful consideration.

### 4.3. Analysis of factors that determine the outcomes of IVF/ICSI treatment in patients with ovarian endometrioma

We found that the number of antral follicles, bFSH, number of retrieved oocytes, 2PN embryos, and 2PN high-quality embryos were significantly higher in pregnant patients with ovarian endometrioma. Furthermore, nonconditional multivariate logistic regression analysis suggested that the number of 2PN high-quality embryos was the only independent predictive factor for clinical outcomes of young patients with ovarian endometriomas, consistent with the results of previous research.^[24–26]^ A total of 95.4% of patients underwent cystectomy before IVF-ET, and 23.4% of patients still had ovarian endometrioma during IVF-ET, showing a high recurrence rate of ovarian endometrioma. In the present study, we found no difference in the endometrioma exist rate during IVF-ET between the successful and failed groups. In the future, we will conduct a randomized controlled study on patient with or without ovarian endometrioma resection before IVF-ET. Because some patients with ovarian endometrioma did not undergo the operation, or some patients underwent cystectomy in other hospitals, we could not compare the relationship between the retrospective American Fertility Society score of EMs and clinical outcomes after IVF-ET.

## 5. Conclusion

In summary, EMs can reduce success clinical outcomes of IVF-ET treatment, while ovarian endometrioma can significantly reduce ovarian function in young patients and affect the treatment outcomes of IVF-ET.

### Author contributions

Feng Zhou and Fanxuan Zhao wrote the manuscript. Feng Zhou and Xiaoying Jin designed the study. Feng Zhou, Fanxuan Zhao and Chao Li analyzed the data. Songying Zhang reviewed the manuscript. All authors contributed to the article and approved the submitted version.

### Acknowledgments

The authors thank Haiyan Zhu for advice on statistics of this manuscript.
